# Schuylkill Valley Veterinary Medical Association

**Published:** 1896-01

**Authors:** Otto Noack

**Affiliations:** Corresponding Secretary


### SCHUYLKILL VALLEY VETERINARY MEDICAL ASSOCIATION.

A MEETING was held at the Board of Health rooms, City Hall, Reading, December 18, 1895. The meeting was called to order at 10.30 A.M., President S. G. Burkholder in the chair. In the absence of Recording Secretary Dr. Fridirici, of Tamaqua, Dr. Noack acted as Secretary. At roll-call the following members responded: Drs. Burkholder, Denver; McCarthy, Fegley, Pottsville; Noack, Reading; Kershner, Fleetwood. The minutes of the last meeting were read and approved.

The Secretary then announced the following applications for membership: Drs. A. R. Potteiger, Selinsgrove; Marsh L. Brackbill, Reading; U. S. G. Bieber, Kutztown. A recess of about ten minutes was then taken that the Trustees might examine the applications. In the absence of Drs. Faughman and Snyder the President appointed Drs. Fegley and McCarthy. Having duly examined the applications Dr. Noack favorably reported the candidates. After balloting the candidates were elected to full membership. After discussion about printing or lithographing the certificates, on motion of Dr. McCarthy and seconded by Dr. Burkholder, it was concluded to have them lithographed and to leave the affair in the hands of Dr. Noack.

Then the matter of meat and milk inspection was entirely discussed, and, in the absence of two members of the Committee on Legislation, Dr. Noack moved that the resolution should be referred to the committee and presented before the next meeting; carried. It was decided to hold the next meeting again at Reading, in the same room, on March 18, 1896. At 12 o'clock a motion was made to adjourn for luncheon and to reassemble at 1.30 P.M.

The afternoon session was called to order at 1.30 P.M. The President called upon Dr. Kershner to read his paper on "Obstetrical Operations." After finishing a lengthy discussion followed by all members present. Dr. Burkholder then read a paper on "Inoculation." The writer showed in his able paper very interesting points of the new treatment of diseases. After discussion the Society, on motion, tendered their thanks to the writer for his valuable contribution.

The following subjects were assigned to be read at the next meeting: "General Pathology of the Lymphatic System," McCarthy; "Mange," Bieber; "Spavin," Brackbill.

Meeting adjourned at 4 o'clock P.M.

OTTO NOACK,
Corresponding Secretary.

